# Enhancing Gluten-Free Bread Quality with Whole-Grain Pearl Millet Flour: A Physicochemical and Sensory Approach

**DOI:** 10.3390/foods15050926

**Published:** 2026-03-06

**Authors:** Bárbara Amorim Silva, Jhony Willian Vargas-Solórzano, Marilia Penteado Stephan, Rosires Deliza, Inayara Beatriz Araujo Martins, Carlos Wanderlei Piler de Carvalho, José Luis Ramírez Ascheri

**Affiliations:** 1 Postgraduate Program in Food Science and Technology, Universidade Federal Rural do Rio de Janeiro, Rodovia Br 465, km 7, Seropédica CEP 23890-000, RJ, Brazil; barbara.amorim.silva@gmail.com; 2 Postdoctoral Program Fundação Arthur Bernardes FUNARBE, Embrapa Agroindústria de Alimentos, Avenida das Américas 29501, Guaratiba, Rio de Janeiro CEP 23020-470, RJ, Brazil; 3 Embrapa Agroindústria de Alimentos, Avenida das Américas 29501, Guaratiba, Rio de Janeiro CEP 23020-470, RJ, Brazil; marilia.stephan@embrapa.br (M.P.S.); rosires.deliza@embrapa.br (R.D.); carlos.piler@embrapa.br (C.W.P.d.C.); 4 Postdoctoral Program Nota 10-FAPERJ, Embrapa Agroindústria de Alimentos, Avenida das Américas 29501, Guaratiba, Rio de Janeiro CEP 23020-470, RJ, Brazil; inayarabeatriz@yahoo.com.br

**Keywords:** cereal milling, dough rheology, baking product, physical property, crumb hardness, overall acceptability

## Abstract

(1) Background: Starch-based breads can closely mimic wheat bread in texture and appearance; however, their nutritional value must be improved while maintaining their inherent bread-like characteristics. The objective of this study was to incorporate whole-grain pearl millet flour (PMF) into a starch-based bread formulation to enhance its dietary fiber and protein content. (2) Methods: The PMF was obtained using a combination of laboratory rollers and hammer mills, as well as appropriate sieves to obtain a particle size ≤ 250 µm. The incorporation of PMF affected the properties of the base flour (BF), dough, and gluten-free bread (GFB). (3) Results: In the BF, setback viscosity was significantly reduced from 6379 to 1354 mPa·s. Similarly, in the freshly kneaded dough, both the elastic and viscous moduli decreased, from 168.3 to 17.8 kPa and from 36.3 to 4.3 kPa, respectively. During fermentation, dough-specific volume increased from 0.76 to 1.73 cm^3^/g. In the GFB, the moisture content decreased from 47.9 to 42.2%, bread specific volume varied from 2.13 to 2.68 cm^3^/g, and crumb hardness increased from 12.8 to 25.3 N. PMF incorporation segmented bread consumers into two preference-based clusters, characterized by lower (1) and higher (2) PMF levels. (4) Conclusions: Incorporating 30% PMF increased the fiber and protein contents of the starch-based bread by 4.9% and 2.2%, respectively, without compromising specific volume (2.56 g/cm^3^) or overall acceptance, which remained comparable to that of a commercial gluten-free bread (7.30 and 6.32 for clusters (1) and (2), respectively).

## 1. Introduction

The use of different starch sources to replace gluten functionality is a strategy already established in the production of gluten-free breads. The most commonly used starches include those from corn, potato, rice, and cassava [1,2,3]. Given that starch-based breads are low in fiber and protein, several studies have incorporated whole flours from cereals or pulses to enhance their nutritional value; however, enrichment with whole flours affects the volume, texture, and color of the bread, especially at high substitution proportions [4].

Whole-grain pearl millet (*Pennisetum glaucum *(L.) R.) is a small cereal with an average grain diameter of 3–4 mm, which has attracted increasing interest due to its nutritional potential. Its endosperm is rich in starch, with granules ranging from 3 to 14 µm and accounting for 63–79% of the grain weight, of which 19–33% corresponds to the amylose fraction. Proteins represent about 9–19% of the grain weight and are primarily located in the endosperm, where prolamins and glutelins predominate, and in the germ, which is enriched in albumins and globulins. In the endosperm, proteins are organized as a continuous matrix embedding starch granules and as discrete spherical protein bodies with diameters ranging from 0.6 to 1.5 µm. In addition, pearl millet is a valuable source of dietary fiber (approximately 7–12%), mainly composed of insoluble fractions such as cellulose and lignin concentrated in the pericarp, while soluble fiber components are distributed within the endosperm cell walls [5].

The incorporation of PMF into a starch-based gluten-free bread resulted in a more compact and moist crumb, with a characteristic cake-like appearance, differing from that typically observed in traditional wheat breads [6,7]. Among the factors associated with this phenomenon are the level of dough hydration, a high degree of starch granule damage in the flour, and the use of finely milled flours with particle sizes < 100 µm [8]. The latter two factors are closely related to the milling system and have important implications for the breadmaking properties of the flour. Together with dough hydration level, they influence dough viscoelasticity during kneading, the expansion kinetics of CO_2_ bubbles during fermentation, and consequently, bread specific volume [9,10]. Gluten-free formulations generally require higher water levels to achieve bread characteristics comparable to wheat breads. This occurs because, in the absence of gluten-forming proteins, dough consistency is primarily governed by starch-water interactions. The addition of hydrocolloids to stabilize these formulations further increases water absorption, as the hydroxyl groups of both hydrocolloids and starches form hydrogen bonds with water [11,12].

Despite the growing interest in pearl millet for gluten-free applications, many studies have not adequately addressed the role of milling conditions in flour preparation as a critical determinant of gluten-free bread quality. Milling-induced starch granule damage, resulting from particle size reduction, increases the exposure of amorphous regions, thereby enhancing water absorption and amylose leaching—key phenomena governing the rheological behavior of gluten-free doughs [13].

In this study, a combination of milling systems and sieving was employed to obtain PMF with particle sizes ≤ 250 µm, while inducing slight starch granule damage [14]. Since bread moisture retention depends on the amount of water and formulation composition used in dough formation [15], as well as on baking conditions, a hydration level of 110% (flour basis) was used to obtain breads with a moisture content of approximately 40%. The formulation composition was primarily modified by incorporating PMF into a starch-based blend, thereby altering starch granule size distribution and protein and fiber contents. These modifications influenced dough rheological properties as well as breadcrumb structure, volume, texture, and sensory acceptability. Accordingly, the objective of this study was to develop gluten-free breads by replacing a starch-based flour with PMF at different levels (0, 10, 30, 50, and 100%). The physical properties of the raw flours, doughs, and breads were characterized, and consumer acceptance of the resulting breads was evaluated.

## 2. Materials and Methods

### 2.1. Materials

Pearl millet grains (*Pennisetum glaucum* (L.) R.), hybrid cultivar ADR9070, were kindly provided by Atto Sementes (Rondonópolis, Brazil). All ingredients used for the preparation of conventional gluten-free breads were purchased from local retail markets in Rio de Janeiro, Brazil: polished rice flour (Tio João-Josapar, Pelotas, Brazil), cassava starch (Aminna Alimentos, Blumenau, Brazil), potato starch (Vitao Alimentos, Curitiba, Brazil), xanthan gum (WeNutri, Santo André, Brazil), dry yeast, table salt, refined sugar, and extra-virgin olive oil. Prior to use, all powdered ingredients were sifted through a 250 µm sieve (Newark, DE, USA) which is equivalent to the American Society for Testing and Materials (ASTM No. 60), complying with ASTM E11 specifications [16] to ensure particle size uniformity. Filtered tap water was used for dough preparation.

### 2.2. Preparation of Whole-Grain Pearl Millet Flour

Foreign materials resulting from grain harvesting were removed using a Clipper Office Tester 400/B (A.T. Ferrell Co., Bluffton, OH, USA). Screen plates with round openings of 2.78 mm and 2.07 mm were used to separate undersized grains, oversized or clumped grains, and residual straw and spikelet fragments.

**Figure 1:**
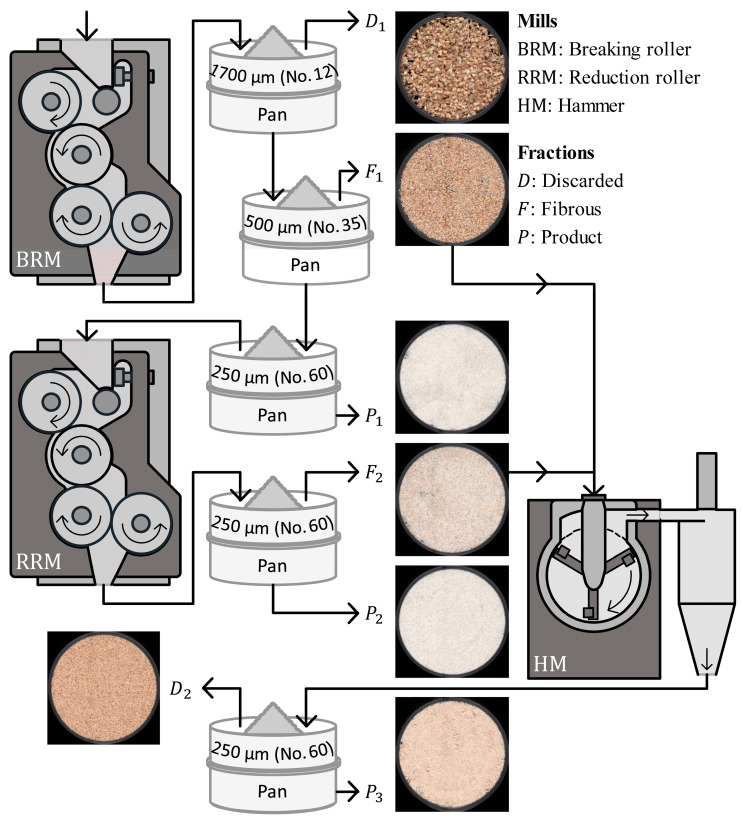
Preparation of whole-grain pearl milled flour (PMF) for gluten-free bread making. Grinder designs were adapted from [17].

The cleaned grains were size reduced to obtain particles ≤ 250 µm using a combination of laboratory-scale roller and hammer mills, followed by sieving, as illustrated in Figure 1. The characteristics of the milling equipment have been previously described by [17]. A sequential milling system was used, consisting of a Quadrumat^®^ Junior laboratory mill (Brabender, Duisburg, Germany) operated without the sieving drum as the breaking roller mill (BRM), followed by the grinding/middlings reduction head of a Quadrumat^®^ Senior laboratory mill (Brabender, Duisburg, Germany) operated without the sifter frame as the reduction roller mill (RRM), and completed by a 3100 laboratory hammer mill (Perten Instruments AB, Huddinge, Sweden) equipped with an 800 µm screen for comminution of insoluble fiber-rich particles. Both roller mills were equipped with four-roll “double-duty” heads operating in three successive roll passes, in which material was sequentially processed between roll pairs 1–2, 2–3, and 3–4 to achieve progressive kernel breakage (BRM) and stepwise middlings reduction (RRM). A combined roller–hammer milling strategy was adopted to achieve fine particle size reduction while controlling mechanical stress on starch granules. Roller milling was used as a first step to promote uniform endosperm fracture and controlled kernel opening, thereby limiting starch granule damage. This preconditioning step reduced both energy demand and impact intensity during subsequent milling, enabling finer size reduction while preserving the functional integrity of starch and protein components. Hammer milling was subsequently applied to efficiently reduce the size of insoluble fiber-rich particles derived from the grain peripheral layers, which require impact forces for effective comminution. This step improved particle size homogeneity and limited the presence of coarse fibrous fragments that could impair dough development and fermentation. Grains were fed into the BRM, with the feed opening set to the third position. The material exiting the BRM was successively sieved to obtain ground product 1 (P1). Particles retained on a 250 µm sieve were subsequently fed into the RRM at an approximate feed rate of 6 kg/h, and the resulting material was sieved to obtain ground product 2 (P2). Material retained on the 250 µm sieve after RRM processing (F2), together with the BRM fraction retained on the 500 µm sieve (ASTM No. 35; F1), was fed into the hammer mill (HM), which was configured with a sieve containing 800 µm openings. The material exiting the HM was sieved to obtain ground product 3 (P3). Particles retained on the 250 µm sieve after HM processing (D2), along with the BRM fraction retained on the 1700 µm sieve (ASTM No. 12; D1), were discarded. The discard fractions D1 and D2 accounted for 0.07 ± 0.01% and 4.91 ± 0.09% of the material, respectively. The milled product streams (P1, P2, and P3) were subsequently blended in a rotary mixer MR10L (Chopin Technologies, Paris, France) for 15 min. The resulting composite flour was labeled as whole-grain pearl millet flour (PMF). This milling process resulted in a flour yield of 93.34 ± 0.10%.

### 2.3. Preparation of Base Flour Blends

A blend of major ingredients (dry basis), consisting of polished rice flour (50%), potato starch (30%), and cassava starch (20%), was prepared using a planetary mixer KEC97A (KitchenAid, Greenville, SC, USA) equipped with a 4.8 L bowl and a flat beater as a mixing device. This blend was partially or fully substituted with PMF at levels of 0, 10, 30, 50, or 100% (dry basis). The major ingredients were mixed at speed 1 (40 rpm) for 5 min, and the resulting dry mixture was designated as the base flour (BF).

### 2.4. Pasting and Hydration Properties of Base Flours

The particle fraction between 106 and 212 μm of the BFs was used to prepare aqueous suspensions (*w*/*w*, dry basis). Pasting properties—namely pasting temperature (PT), peak viscosity (PV), trough viscosity (TV), gelation viscosity (GV), breakdown viscosity (BV), and setback viscosity (SV)—were measured in duplicate using 28 g of a 9.21% aqueous suspension heated under constant stirring (160 rpm) in a Rapid Visco Analyzer RVA Series 4 (Newport Scientific Pty Ltd., Warriewood, Australia), according to [18]. Briefly, the suspension was equilibrated at 25 °C for 2 min, heated to 95 °C at a rate of 14 °C/min, maintained at this temperature for 3 min, and subsequently cooled to 25 °C at the same rate. Hydration properties, including the water solubility index (WSI) and water absorption index (WAI), were determined in quadruplicate using 11 g of a 9.09% aqueous suspension, following the method described by [19]. Briefly, the suspension was maintained at 25 °C for 30 min in an NT 232 Dubnoff water bath (Novatecnica, Piracicaba, Brazil) and then centrifuged at 9961× *g* for 15 min at 25 °C using a Universal 320R refrigerated centrifuge (Hettich, Tuttingen, Germany). The supernatant was collected, and its aqueous phase was oven-dried at 105 °C to obtain the dry residue (soluble solids), which was used to calculate the WSI in g soluble solids per 100 g of dry solids (g ss/100 g ds). Conversely, the aqueous phase retained in the precipitate (insoluble solids) was used to determine the WAI in a g of absorbed water per g of insoluble solids in the sample (g w/g is).

### 2.5. Preparation of Functional Additives and Yeast Activation

Xanthan gum (2% of BF) was manually dispersed in olive oil (3% of BF) to enhance oil droplet stability during dough kneading. Separately, dry yeast (1% of BF) and refined sugar (2% of BF) were manually dissolved in tempered water at 37 °C, using 25% of the total water to be added. This mixture was then incubated for 5 min in a fermentation-controlled chamber AC20T (Metalúrgica Venâncio Ltda, Venâncio Aires, Brazil) at 37 °C and 85% relative humidity.

### 2.6. Mixing Procedure and Dough Formation

Minor ingredients were added to the BF contained in the mixing bowl in the following sequence: functional additives, table salt (1.5% of BF), and activated yeast. After the addition of each ingredient, the dough was intermittently kneaded at speed 2 (50 rpm) for 3 min. The remaining water (75%) was then gradually incorporated under continuous kneading at speed 4 (95 rpm) for 3 min. Subsequently, the kneading speed was progressively increased from speed 6 (130 rpm) to speed 10 (195 rpm) until a smooth and homogeneous dough consistency was obtained. The total dough development time was approximately 20 min. This straight dough mixing procedure was adapted from standardized breadmaking guidelines [20] and designed in accordance with the general principles for breadmaking tests outlined in [21]. After mixing, 1 kg of dough was transferred into an aluminum mold (22 × 11 × 10 cm) and shaped by gentle tapping.

### 2.7. Dough Rheological Properties

Dough samples were prepared following the breadmaking procedure described above, with the exception of yeast addition to avoid structural changes associated with gas production during fermentation. The viscoelastic properties of the dough were evaluated by small-amplitude oscillatory shear measurements, focusing on the elastic (G′) and viscous (G″) moduli. Measurements were performed in duplicate using a modular compact rheometer MCR 92 (Anton Paar GmbH, Graz, Austria) configured with parallel plate geometry (PP25) and an environmental measuring chamber to minimize moisture loss during testing. Dough samples were carefully loaded between the plates to minimize structural disruption; excess material was trimmed, and a fixed gap of 1 mm was applied. Oscillatory frequency sweep tests were conducted at 25 °C over a frequency range of 0.1–10 Hz using a constant shear strain of 0.015%, previously determined to fall within the linear viscoelastic region by strain sweep tests. This experimental approach is widely used to characterize the viscoelastic behavior and structural strength of gluten-free and starch-based dough systems [22,23]. The G′ and G″ moduli were calculated from the raw rheological data using RheoCompass^®^ software, version 1.24.510 (Anton Paar GmbH, Graz, Austria).

### 2.8. Fermentation and Baking Procedures

The molded dough was transferred to the fermentation-controlled chamber used for yeast activation. Fermentation was considered complete when the dough reached approximately twice its initial volume or approached the height of the mold [24,25]. Under these conditions, fermentation time decreased from 65 to 55 min as the level of BF substitution with PMF increased, indicating faster dough expansion at higher PMF contents. The fermented dough was then immediately baked in a turbo oven VM020141 (Metalúrgica Skymsen Ltda, Brusque, Brazil) preheated to 200 °C. Baking was carried out for 1 h, after which the loaf was cooled in a conditioned environment at 20 °C for 3 h and subsequently packaged in a polyethylene bag. After 16 h of storage, the bread was sliced transversely using a bread slicer FP-12S (G.Panis Ltda, Caxias do Sul, Brazil) to obtain 16 slices of approximately 10 mm thickness, with the lateral end slices discarded. Five experimental gluten-free breads were produced according to the PMF substitution level (GFB-0, GFB-10, GFB-30, GFB-50, and GFB-100). For comparison, wheat bread (WB) was prepared under the same processing conditions, in accordance with the general guidance provided by [21] for breadmaking tests, and only after the gluten-free breads to avoid cross-contact.

### 2.9. Determination of Dough and Bread Specific Volume

Three cylindrical containers of known mass ($m_{c}$) and volume ($V_{c}$) were used to monitor dough expansion during fermentation, assuming uniaxial (vertical) expansion, according to [9]. The dough was transferred into the cylinders using a pastry bag fitted with a confectioner’s nozzle until the containers were completely filled. The dough was then manually compacted, leveled with a spatula to match the cylinder rim, and transferred to the fermentation chamber. The mass of each cylinder-dough system ($m_{c+d}$) was recorded at the beginning of fermentation and at 20 min intervals thereafter, following the removal of fermented dough exceeding the cylinder height. Dough specific volume (DSV) was calculated according to Equation (1), where t represents the time at which the mass was recorded.

(1) $$\mathrm{DSV}={\left(\frac{V_{c}}{m_{c+d}-m_{c}}\right)}_{t}$$

For bread measurements, a separate cylindrical container of known mass ($m_{c}$) and volume ($V_{c}$) was used to freely accommodate a bread slice of known mass ($m_{b}$). The bread slice volume was determined in triplicate using the seed displacement method [26], in which pearl millet seeds were allowed to fall freely from a height of 25 cm to ensure uniform filling. The bread specific volume (BSV) was calculated using Equation (2):

(2) $$\mathrm{BSV}\;=\;\frac{V_{c}}{m_{b}}\left(\frac{m_{1}-m_{2}+m_{b}}{m_{1}-m_{c}}\right)$$

where $m_{1}$ is the mass of the cylinder filled with seeds, and $m_{2}$ is the mass of the cylinder containing both the bread slice and the seeds.

### 2.10. Determination of Bread Moisture Content

Fresh bread slices taken from the central portion of each loaf were used to determine moisture content in triplicate. The mass of the bread slice ($m_{b}$) was recorded, after which the crust was carefully removed using a knife. The mass of the crust ($m_{ct}$) was then recorded, and the moisture contents of the crust ($M_{ct}$) and crumb ($M_{cb}$) were determined in accordance with AOAC Official Method 925.09 [27]. The overall bread moisture content ($M_{b}$) was subsequently calculated by applying a mass balance that considers the moisture content and mass fraction of crumb and crust regions, as described in Equation (3):

(3) $$M_{b}=M_{cb}-\frac{m_{ct}}{m_{b}}\left(M_{cb}-M_{ct}\right)$$

### 2.11. Proximate Composition Analysis of Bread

Fresh breadcrumbs (200 g) were vacuum-dried in an SL-104/12 (SOLAB, São Paulo, Brazil) at 50 °C under 668 mmHg, with periodic removal of condensed water, until crumb moisture content decreased below 12%, as estimated from mass loss. The dried crumb was size reduced using a 3100 hammer Lab Mill (Perten Instruments AB, Huddinge, Sweden) configured with an 800 µm sieve. Proximate composition of the resulting crumb flour was determined in duplicate according to AOAC methods [27]: moisture (925.09), ash (923.03), total nitrogen (2001.11), lipids (945.38), and dietary fiber (985.29). Protein content was calculated as total nitrogen × 6.25, and available carbohydrates were determined by difference. Crumb flour moisture ranged from 4.62% to 11.70%. Proximate composition data, expressed on a dry basis, are reported in Supplementary Table S1 and were used to estimate base flour and dough formulations.

### 2.12. Crumb Structure and Microstructural Analysis

Bread slice images were obtained using a 1240U Perfection Office Scanner (Epson, Seiko, Suwa, Japan). The slices were positioned within a standardized scanning area of 12.5 × 11.5 cm, and image acquisition was performed using PhotoFiltre image-editing software, version 7 under fixed acquisition settings (exposure 3, gamma 1, highlight 200, shadows 24), following [28]. The bread’s enlargement was incorporated into the scanned images as oven spring (OS), calculated as the % increase in the BSV since the end of fermentation. Crumb microstructure was examined by scanning electron microscopy (SEM). Dried crumb samples were prepared as cylindrical specimens (5 mm diameter × 3 mm height) and analyzed without sputter coating in a TM3000 benchtop SEM (Hitachi HighTechnologies Corporation, Tokyo, Japan), which operates in a low-vacuum mode. Samples were mounted on aluminum stubs using double-sided conductive adhesive tape. Following the manufacturer’s recommendations, sample height was adjusted to maintain a gap of approximately 1 mm between the specimen height gauge and the topmost sample surface. Images were acquired using TM3000 software (version 02-01), which automatically regulates emission current through brightness and contrast control. Observations were performed at an accelerating voltage of 5 kV. An air cell wall was selected as the observation point, and micrographs were recorded at a magnification level of ×500. Automatic adjustment modes were applied for focus, brightness, and contrast. Under these conditions, the detector working distance corresponded to D4.2–D4.5.

### 2.13. Crumb Texture Profile Analysis

Cylindrical crumb specimens were prepared using a stainless-steel ring (26.6 mm internal diameter). For each treatment, the mass and thickness of 15 specimens were recorded and then analyzed with a TA-XT2 Plus texturometer (Stable Micro Systems, Godalming, UK) configured with a 5 kg load cell. The P/36R cylindrical aluminum probe was used to deform the sample in two consecutive compression cycles to 50% of the original sample height at a test speed of 1 mm/s, following the procedure described by [29]. Force acquisition was initiated upon exceeding a trigger force of 0.049 N, with a 5 s interval between the first and second deformation cycles. Texture parameters—hardness (HA, N), cohesiveness (CO), springiness (SP), and chewiness (CH, N)—were calculated from the force-time curves using the Exponent software (version 4.0.13.0) over a total test duration of 30 s.

### 2.14. Crumb Color Measurement

Instrumental crumb color was measured in quadruplicate using a Color Quest XE colorimeter (Hunter Lab., Reston, VA, USA). Prior to measurements, the instrument was allowed to warm up and was standardized through the EasyMatch QC software, version 1.4 using manufacturer-supplied white and black reference tiles, thereby defining the upper and lower limits of the color scale under the same illuminant, observer angle, and measurement geometry applied to the samples. Central crumb sections (~50 mm in height) were placed over the instrument’s 2.54 cm diameter measurement aperture. Measurements were performed in reflectance mode with the specular component excluded (RSEX) to minimize the influence of crumb surface heterogeneity. The sample was then exposed to illuminant D65 using the CIE 10° standard observer, and color coordinates were expressed in the CIELAB system as L* (lightness, 0 = black to 100 = white), a* (from green (−80 to 0) to red (0 to +100)); and b* (from blue (−100 to 0) to yellow (0 to +70)).

### 2.15. Consumer Acceptance Test

A total of 100 consumers (18–70 years old; female/male ratio 57/43) were randomly and spontaneously recruited at the Carlos Henrique Simonsen Shopping Center (Barra da Tijuca, Rio de Janeiro, Brazil) to participate in an overall acceptance test. The evaluation was conducted in a separate, covered, quiet, and well-lit booth, consistent with recommendations for central location tests and consumer hedonic evaluations in controlled areas [30]. This approach allows efficient recruitment of a large and heterogeneous consumer population under controlled serving conditions while maintaining realistic consumption contexts for acceptance measurement. Five experimental GFBs and one commercial sample (GFB-C) were evaluated using a nine-point hedonic scale (1 = dislike extremely; 9 = like extremely). Each participant received one-quarter of a bread slice, coded with three-digit random numbers. Samples were served monadically at room temperature, and the presentation order was balanced according to a Williams’ Latin square design. Prior to tasting, participants were informed that, although the products were formulated as gluten-free, they were manufactured in facilities that handle wheat and therefore might contain traces of gluten. During tasting, participants were instructed to cleanse their palates with water between samples. After sensory evaluation, participants completed a questionnaire addressing sociodemographic characteristics and gluten-free bread consumption habits. Sociodemographic data are provided as Supplementary Table S2.

### 2.16. Experimental Design and Statistical Analysis

The partial or total incorporation of PMF into the BF was structured as a single-factor experimental design with five PMF substitution levels (0, 10, 30, 50, and 100%, dry basis). Analyses of variance (ANOVA) were applied to the mean values of all response variables obtained from two independent experimental replicates.

Overall acceptance data were analyzed via two-way ANOVA, considering bread type (BT) as a fixed factor and consumer as a random factor. Because the random effect was significant, hierarchical cluster analysis was performed using Euclidean distance and Ward’s agglomerative method to identify consumer preference segments. To assess the influence of preference segmentation and prior gluten-free bread consumption on product acceptance, the factors of cluster and consumption habit were included in the ANOVA model.

When significant effects were detected (*p* < 0.05), mean comparisons were conducted using Tukey’s Honestly Significant Difference (HSD) test. Student’s *t*-test was used to compare mean acceptance scores between consumer segmentation approaches. Additionally, internal preference mapping was performed by Principal Component Analysis (PCA) to visualize relationships between samples and consumer preferences. All statistical analyses were carried out using STATISTICA software, version 12 (StatSoft Inc., Tulsa, OK, USA).

## 3. Results and Discussion

### 3.1. Pasting and Hydration Properties of Base Flour

**Figure 2:**
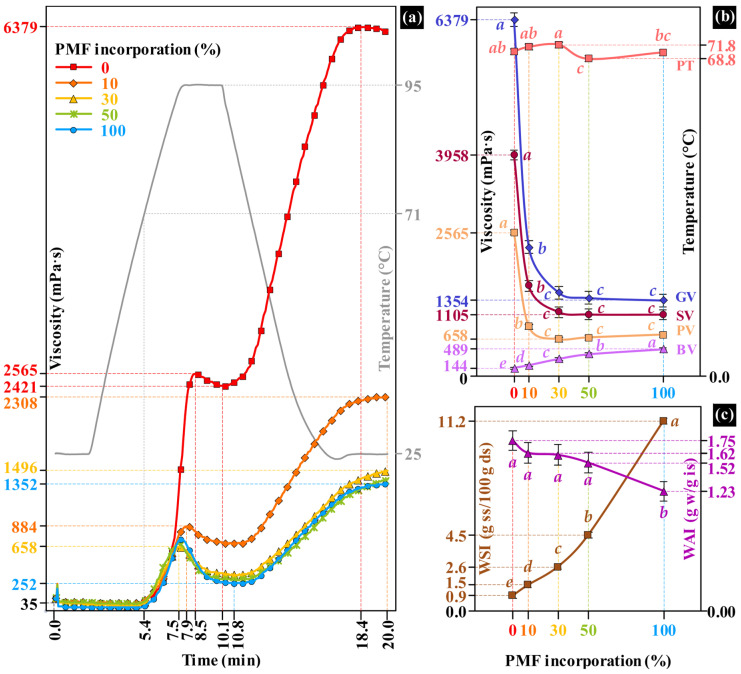
Pasting and hydration responses of a starch-based flour blend as affected by whole-grain pearl millet flour (PMF) incorporation. (**a**) RVA viscosity profiles; (**b**) pasting properties: peak viscosity (PV), gelation viscosity (GV), breakdown viscosity (BV), and setback viscosity (SV); (**c**) hydration properties: water solubility index (WSI) and water absorption index (WAI). Different lowercase letters within the same property differ significantly (Tukey’s test, *p* < 0.05).

The pasting onset of the BF occurred, on average, at approximately 5.4 min (Figure 2a). The addition of PMF had only a minor effect on PT, which ranged from 68.8 °C at 50% PMF to 71.8 °C at 30% PMF (Figure 2b and Table S3). In contrast, PV, TV, and GV viscosities were significantly altered, leading to marked changes in BV and SV viscosities (Figure 2a,b). The PV occurred 0.6–1.0 min earlier, whereas the TV was delayed by approximately 0.7 min. Because no gelation peaks were detected for PMF-containing suspensions within the 20 min RVA test, the final viscosity was considered equivalent to GV. Incorporation of 10% PMF caused pronounced reductions in PV, TV, and GV (65.5%, 71.5%, and 63.8%, respectively; *p* < 0.05). Similar decreases in pasting viscosities have been reported when finger millet flour was incorporated into wheat flour systems in the range of 0–30% [31]. Concomitantly, BV increased significantly from 144 to 489 mPa·s, whereas SV decreased from 3958 to 1102 mPa·s (*p* < 0.05). At PMF addition levels ≥ 30% PMF, changes in BV and SV were less pronounced and no longer statistically significant (*p* ≥ 0.05). The RVA profile of 100% PMF contrasted with [32], where an absence of a sharp PV and, consequently, a low BV was observed.

The reduction in paste viscosity may be attributed to physical and molecular constraints imposed by PMF incorporation. Fine pericarp fragments (≤250 µm), characterized by a needle-like morphology and enriched in insoluble fibers, likely hinder starch granule swelling during gelatinization and restrict reassociation of leached polymers during cooling [18]. In parallel, increased levels of lipids, proteins, and free phenolic compounds may promote non-covalent interactions and complex formation with solubilized amylose during hydration and thermal processing [33,34], thereby further suppressing SV, particularly at low PMF inclusion (10%). The relatively low holding temperature applied in the RVA protocol (25 °C) may also have contributed to the high SV value observed in the control formulation.

SV is closely related to starch retrogradation and bread staling behavior. Lower SV values are generally associated with reduced crumb firming during storage [35]. The stabilization of SV observed at PMF levels ≥ 30% may result from the combined emulsifying and hydrocolloid-like effects of lipids, proteins, and soluble fiber, which can stabilize the starch gel matrix and offset the disruptive role of insoluble fiber, as previously observed in a standard wheat-based dough formulation containing emulsifiers and hydrocolloids [36].

WSI increased progressively with PMF incorporation (Figure 2c and Table S3), rising from 0.9 to 11.2 g soluble solids per 100 g of flour (dry basis). This behavior may be attributed to the leached soluble fraction of dietary fibers and proteins, as well as free phenolic compounds and low molecular weight carbohydrates contributed by pearl millet. The presence of free sugars is particularly relevant for fermentation performance. Elevated α-amylase activity in pearl millet may promote saccharide release [37], enhancing flour solubility and fermentable substrates. Moreover, soluble dietary fibers are known to chelate mineral elements and form charged polysaccharide mineral complexes [38]. At 100% PMF substitution, this mechanism likely increased the density of ionic sites within the system, thereby enhancing electrostatic interactions with charged amino acid residues of proteins embedded in the starch matrix. These interactions may facilitate protein solubilization, contributing to the overall increase in WSI.

WAI (g of absorbed water per g of insoluble solids in the flour) decreased from 1.75 to 1.23 g/g, the reduction being only significant at 100% PMF substitution (*p* < 0.05; Figure 2c and Table S3). The reduction in WAI as a function of formulation composition may be attributed to the combined effects of a low proportion of damaged starch (<0.5%), reduced protein content, the presence of fine insoluble fiber particles, and lipid sources incorporated during the initial mixing stage, all of which limit water uptake and retention by the flour matrix [39,40]. As a whole-grain flour, PMF contains endogenous proteins, lipids, and a dietary fiber fraction dominated by insoluble components (>86%, Table S1). Despite the higher protein concentration as PMF content increased, the simultaneous increase in insoluble dietary fiber and lipid contents likely constrained the functional contribution of proteins to water absorption by reducing the accessibility of hydrophilic and charged amino acid residues. In addition, lipids may coat starch and protein surfaces, limiting capillary water uptake and restricting matrix swelling, which collectively contributed to reducing WAI.

In addition, mechanical grinding can induce structural damage in finely milled insoluble fibers, altering particle morphology and surface characteristics that impact water adsorption. This may have occurred during the size reduction of the PMF fibrous fractions (F1 and F2, Figure 1). According to [10], hammer-milled flours from white proso millet exhibited a lower water-holding capacity than other milling systems, probably because more milling-induced structural damage to fibers occurred.

On the other hand, insoluble dietary fibers exhibit slower hydration kinetics than starch and soluble fibers because their rigid, highly crosslinked cell wall structure limits water absorption and swelling [4]. Consequently, under the hydration conditions used for WAI determination, water uptake is preferentially governed by starch granules.

At ambient temperature, water absorption is largely governed by starch granule size distribution and starch blend composition, which, according to the mean diameter (MD) and the number of granules provided by each starchy ingredient, the total granular surface area will be defined. A greater granular surface area, as occurs in B-type starches, provides a higher ability to swell [41]. By using 100% PMF, the extremely small granules provided by rice starch (4.7 µm MD) and the larger granules provided by potato (42.3 µm MD) and cassava (23.2 µm MD) starches were replaced by slightly smaller granules from pearl millet starch (8.5 µm MD) [5,42]. Considering these MD values, the change in the BF composition from 0 to 100% PMF caused approximately a 30% decrease in the total granular surface area and consequently reduced the overall WAI.

### 3.2. Dough Viscoelastic Properties

**Figure 3:**
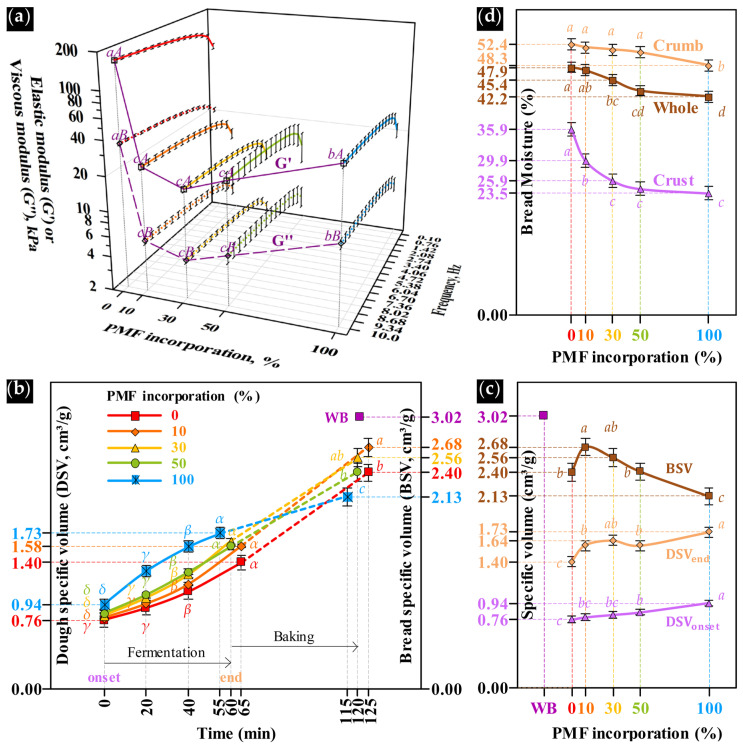
Physical changes before and during fermentation of doughs and after cooling of breads. (**a**) Viscoelastic properties of doughs before fermentation; specific volume of doughs and breads: (**b**) as a function of time and (**c**) as a function of PMF incorporation; (**d**) bread moisture distribution. PMF: whole-grain pearl millet flour, WB: wheat bread. Different lowercase letters within the same curve, or different uppercase letters at the end of fermentation or baking, differ significantly (Tukey’s test, *p* < 0.05).

The effect of PMF incorporation on gluten-free dough consistency was assessed through oscillatory rheology by monitoring the G′ and G″ moduli. Across the tested frequency range (0.1–10 Hz), both moduli exhibited a slight frequency dependence (Figure 3a and Table S4), with G′ consistently exceeding G″, indicating a predominantly elastic, weak gel-like structure irrespective of formulation [15,43]. The starch-based dough (0% PMF) exhibited the highest viscoelastic moduli (G′ = 168.3 kPa; G″ = 36.3 kPa at 10 Hz). Partial substitution with PMF (10–50%) markedly reduced both G′ and G″ to statistically similar levels (*p* ≥ 0.05), with G′ ranging from 17.8 to 24.5 kPa and G″ from 4.3 to 5.6 kPa at 10 Hz. In contrast, the dough formulated with 100% PMF displayed a significant increase in both moduli (G′ = 42.5 kPa; G″ = 10.0 kPa; *p* < 0.05), suggesting partial recovery of structural rigidity. These rheological responses are strongly influenced by formulation water content and matrix composition. Ref. [44] reported substantially lower moduli (G′ < 5 kPa; G″ < 1 kPa) for a 100% millet dough prepared at a higher hydration level (160%), highlighting the dominant role of water content in controlling gluten-free dough rheology.

The pronounced reduction in dough stiffness, evidenced by the sharp decrease in both moduli following only 10% PMF incorporation, may be attributed to the dispersion of insoluble fiber particles within the starch-based matrix. These fibers disrupt starch-starch interactions and weaken the continuity of the viscoelastic network. Given the limited compatibility between insoluble fiber particles and the starch phase, dough extensibility and tensile strength are reduced, reflecting a loss of elastic behavior [45]. The decrease in stiffness observed between 10 and 50% PMF substitution was statistically similar (*p* ≥ 0.05). In contrast, the partial recovery of G′ and G″ at 100% PMF substitution is likely related to the protein content increment, which may enhance matrix cohesion through protein-mediated interactions. This behavior is consistent with reported effects of protein type on the rheological properties of corn starch-protein doughs under optimal hydration conditions [15].

### 3.3. Physical Changes During Fermentation and Baking

DSV at the onset of fermentation ranged from 0.76 to 0.94 cm^3^/g as a function of PMF incorporation, and this trend was observed throughout the fermentation interval (Figure 3b, left axis, Figure 3c, and Table S4). The protein content gradually introduced by PMF likely contributed to dough expansion under hydrated conditions, as protein-bound water increases matrix plasticity. Only doughs with 10–30% PMF doubled their initial volume by the end of fermentation (~101% increase), whereas control and ≥50% PMF doughs reached only ~85% expansion, likely due to low protein content in the former and fiber-induced structural disruption in the latter. Fermentation time decreased from 65 to 55 min with increasing PMF, accompanied by a rise in final DSV from 1.40 to 1.73 cm^3^/g. The shorter fermentation and higher DSV observed in PMF-containing doughs are consistent with the higher endogenous enzyme activity of pearl millet [37], which could have contributed to increased fermentable sugars for yeast metabolism and CO_2_ production [46].

**Figure 4:**
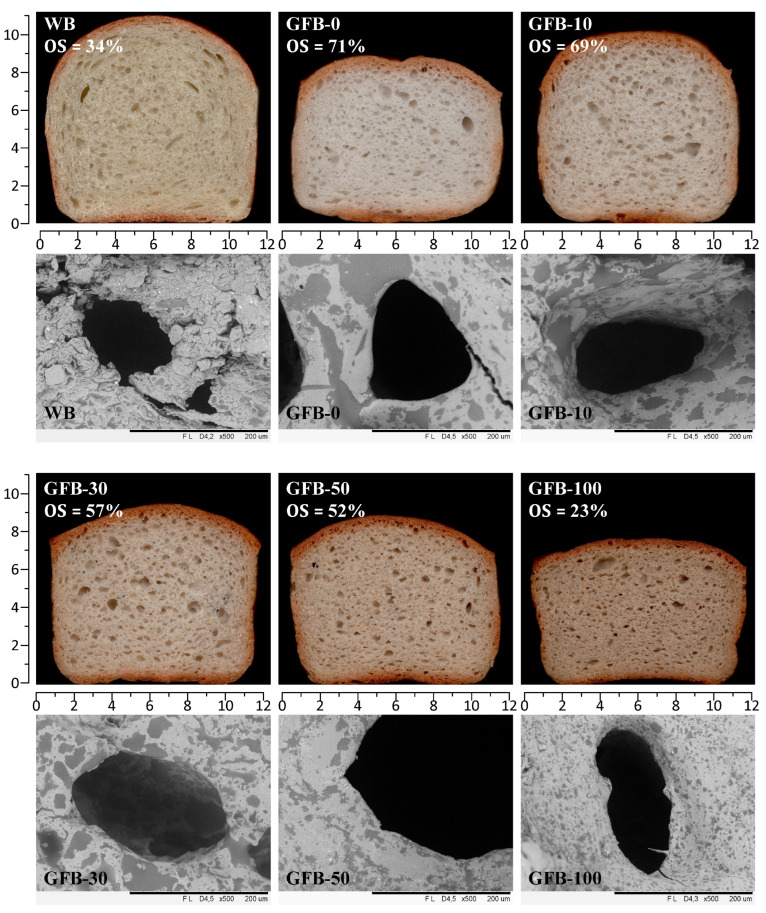
Scanned images and crumb micrographs for wheat bread (WB) and gluten-free breads (GFB) as affected by whole-grain pearl millet flour incorporation (in %). OS: oven spring. Area of scanned images: 12.4 × 11.2 cm. Area of micrographs: 339.7 × 254.8 μm.

During the early baking stage, yeast activity progressively declines above ~43 °C and is fully inactivated near 55 °C. As dough temperature approaches ~60 °C, starch gelatinization initiates and α-amylase activity is maximized between 60 and 70 °C, promoting rapid sugar release and contributing to oven spring [47]. In formulations containing ≥30% PMF, residual sugars likely intensified Maillard reactions during baking above 115 °C, resulting in darker crumb and crust coloration (Figure 4: GFB-30, GFB-50, and GFB-100) and flavor development [47,48]. This browning effect was reflected by lower L* values and positive a* and b* coordinates, as reported in Table S1 for these formulations.

After baking, BSV reflects the dough’s ability to retain CO_2_ within the stabilized crumb structure. PMF incorporation significantly affected BSV (Figure 3b, right axis, Figure 3c, and Table S4). GFB-10 and GBF-30 exhibited the highest BSV values (2.68 and 2.56 cm^3^/g, respectively; *p* < 0.05), although still lower than WB (3.02 cm^3^/g). GFB-0 and GFB-50 showed comparable volumes (~2.40 cm^3^/g, *p* ≥ 0.05), whereas GFB-100 resulted in the lowest BSV (2.13 cm^3^/g).

The highest BSV observed for GFB-10 and GFB-30 is consistent with [49], who reported enhanced gas production—reflected by higher DSV after 40 min of fermentation—in rice flour-corn starch doughs containing 10% fine oat or bamboo fiber particles. This enhanced fermentative activity translated into a significantly higher BSV compared to the fiber-free control. In contrast, although formulations containing ≥50% PMF exhibited higher fermentative activity, they displayed reduced gas retention, as evidenced by lower BSV. This behavior may be attributed to the accumulation of fine insoluble fiber particles, which disrupt interfacial stability, weaken the viscoelastic matrix, and promote gas cell coalescence and collapse during baking [4]. In addition, bread volume is adversely affected in dough formulations containing lipid levels above 6% [40], as observed for GFB-100 (Table S1), where excess lipids are known to impair gas cell stabilization and limit bread expansion.

Bread moisture decreased with PMF incorporation, from 47.9% to 42.2%. Similar trends were observed for crumb and crust moisture, which declined from 52.4% to 48.3% and from 35.9% to 23.5%, respectively (*p* < 0.05, Figure 3d and Table S4). This behavior may be attributed to starch dilution—the main water-binding component in fermented doughs—as available carbohydrate content decreased from 85.26% to 61.64% (Table S1). Beyond starch quantity, intrinsic starch properties may influence baking performance and crumb structure, such as granule size distribution, crystalline organization, and the extent of amylose leaching during gelatinization. Amylose plays a central role in stabilizing crumb cell walls by reinforcing the starch gel network and enhancing gas retention [50]. During baking, starch gelatinization entraps water within this viscoelastic matrix, whose cell wall thickness varies from 20 to 200 µm [51]. However, reduced amylose leaching, lower crystallinity of ghost granules, the presence of finely milled insoluble pericarp fibers, and the limited structuring role of proteins collectively weaken the starch gel network and increase the permeability of crumb cell walls. Consequently, water vapor diffuses more readily through the crumb structure, resulting in higher moisture loss during baking of PMF-enriched formulations. Although PMF incorporation increased protein content, it did not effectively reinforce the crumb matrix. Thus, starch functionality remained the dominant factor governing baking performance. As a result, starch dilution prevailed, leading to reduced final bread moisture.

### 3.4. Structure and Microstructure of Breads

Figure 4 presents scanned slices of the experimental GFBs, with WB included as a reference. Clear differences in crumb height were observed, reflecting variations in oven spring among formulations (23–71%). Dome formation, commonly used as an indicator of the strength of the dough system because of its association with improved volume development and crumb structure [47], was most pronounced in GFB-10 and closely resembled that of WB. All PMF-containing breads exhibited some degree of dome formation; however, its intensity progressively decreased at higher substitution levels, being least evident in GFB-100, consistent with its lower BSV.

WB exhibited a yellowish crumb with a well-developed cellular structure, characterized by small, spherical air cells near the center and larger, elongated cells toward the periphery, together with a thinner and lighter crust compared with all GFBs. At the microstructural level, WB crumb displayed rough air cell walls composed of aggregated and deformed gelatinized starch granules, whose granular integrity was largely preserved by the continuous gluten network, resulting in irregular and well-defined cell borders. In contrast, GBFs showed a gradual darkening of crumb and crust with increasing PMF incorporation, as evidenced by the decrease in crumb L* values from 68.90 to 56.96 (Table S1). The air cell distribution differed markedly from that of WB, lacking the characteristic spiral pattern due to the ease with which the wheat dough can form coiling layers during kneading. Larger air cells were observed in GFB-10 and GFB-30, whereas higher PMF levels (≥50%) resulted in a more compact crumb structure dominated by smaller air cells. Additionally, crust thickness progressively decreased as PMF substitution increased.

Microstructural observations further revealed that the crumb surface of GFB-0 contained elongated dark regions relative to lighter areas. With increasing PMF addition, these dark regions became progressively fragmented. The smooth appearance of both light and dark regions observed in the GFB crumbs represents a distinctive structural characteristic arising from the absence of a gluten-like network, wherein crumb organization is governed primarily by hydrocolloid–biopolymer interactions. This microstructural aspect can, therefore, be attributed to the stabilizing effect of the added xanthan gum, which promotes uniform biopolymer distribution and cohesive matrix formation within the air cell walls [52]. Moreover, glucuronic acid residues in xanthan gum may ionize, creating negative charges to further facilitate interactions with mineral elements [53], contributing to the observed microstructural smoothness.

The dark regions may be interpreted as melted and aggregated ghost starch granules, whereas the lighter regions are likely composed of leached amylose forming macromolecular complexes with lipids, proteins, and dietary fiber. As PMF incorporation increased the concentration of these biopolymers, their collective emulsifying and interfacial stabilization capacity was enhanced. Consequently, the extent of starch granule melting and aggregation was reduced, resulting in smaller and more finely dispersed dark regions within the crumb matrix.

### 3.5. Texture Profile Analysis

**Figure 5:**
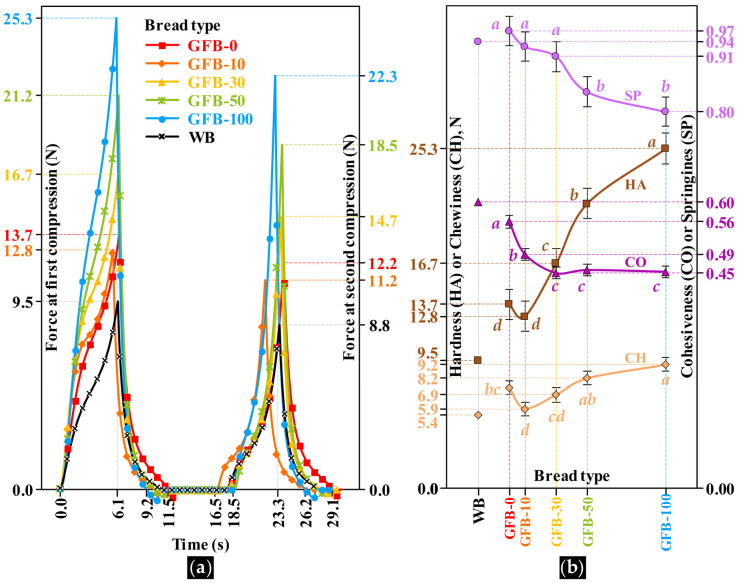
(**a**) Texture profiles of gluten-free bread (GFB) crumbs as affected by whole-grain pearl millet flour incorporation (PMF, %); (**b**) change in texture parameters: hardness (HA), chewiness (CH), cohesiveness (CO), and springiness (SP). Values with different lowercase letters within a parameter differ from each other according to the Tukey test. Different lowercase letters within the same parameter differ significantly (Tukey’s test, *p* < 0.05).

The breadcrumb exhibited distinct mechanical behavior, as evidenced by the progressive increase in the area under the force-time curves with increasing PMF content in the GFBs. WB exhibited the smallest area under the curve, indicating a softer and more elastic crumb compared with all GFBs (Figure 5a). HA of the GFB samples ranged from 12.8 to 25.3 N, values that were substantially higher than that of WB (9.5 N). The softest crumbs were observed in GFB-0 and GFB-10, which were statistically similar (*p* ≥ 0.05; Figure 5b), whereas breads containing higher levels of PMF exhibited significantly increased resistance to compression (*p* < 0.05). This increase in HA is consistent with the structural features observed in Figure 4 for GFBs containing ≥30% PMF, which exhibited fewer air cells and thicker cell walls, resulting in a more compact crumb structure. Notably, the maximum hardness recorded for GFB-100 remained below the average human chewing force reported for whole wheat bread (~30 N), suggesting acceptable mastication properties in the experimental breads.

Analysis of the force-time curves further revealed differences in elastic recovery behavior. The first compression peak occurred at approximately 6.1 s for all samples, including WB, with the exception of GFB-10, which reached peak force slightly earlier. Greater variability was observed during the second compression cycle: while WB exhibited a second peak at 23.3 s, GFB-10 showed a noticeably delayed response.

These temporal shifts reflect differences in SP, which decreased from 0.97 to 0.80 as PMF content increased (Figure 5b). Notably, the SP values of GFBs containing ≤30% PMF were statistically similar to each other (*p* ≥ 0.05) and comparable to that of WB (0.94), whereas higher PMF incorporation led to a significant loss of elastic recovery.

The duration of the compression cycles varied among bread types and influenced CO. The first compression cycle ended between 9.2 and 11.5 s, whereas the second cycle occurred between 26.2 and 29.1 s. Relative to the WB profile, GFB-0 showed a delayed response, while GFB-10 exhibited an earlier completion of the cycle, both indicative of more cohesive crumbs (0.56 and 0.49, respectively), although still lower than that of WB (0.60). From 30% PMF incorporation onward, CO decreased and remained similar among formulations (0.45, *p* ≥ 0.05), suggesting that higher PMF levels limited the ability of the crumb structure to recover after deformation.

CH, which ranged from 5.9 to 9.2 N and did not differ significantly between GFB-10 and GFB-30 (*p* ≥ 0.05). GFB-10 exhibited CH values closest to those of WB (5.4). The progressive increase in CH and HA, together with the concomitant reduction in CO and SP as PMF content increased (Figure 5b), may be attributed to compositional changes in the crumb (Table S1) and the reduced moisture levels (Figure 3c). These factors likely contributed to firmer and less elastic crumbs, thereby increasing resistance to mastication.

In comparison with previously reported breads formulated with 100% PMF, ref. [7] reported lower HA values, which may be attributed to differences in bread composition, notably higher moisture and lower dietary fiber contents. In contrast, lower CO and SP values have been reported by [54], suggesting that variations in milling processing conditions markedly influence the PMF fiber composition and mechanical behavior of PMF-based breads.

### 3.6. Consumer Acceptance Results

Based on gender and prior consumption of GFB (Table S2), the participants comprised 57 females (Yes, *n* = 33; No, *n* = 24) and 43 males (Yes, *n* = 25; No, *n* = 18). Most participants were 26–35 years old, had completed higher education, and reported a monthly income between US$280 and US$560. To further explore consumer heterogeneity, cluster analysis identified two distinct preference segments: cluster (1), *n* = 53, and cluster (2), *n* = 47. When stratified by prior GFB consumption, significant differences were observed in age and income distribution, with regular GFB consumers being more frequently represented in the 36–45 year age group and higher income brackets.

Table 1 summarizes the mean acceptance scores of the evaluated GFBs, both overall and segmented according to consumer preference clusters and prior consumption habits. Overall, GFB-C and GFB-30 achieved the highest acceptance scores, differing significantly (*p* < 0.05) from GFB-0, GFB-10, and GFB-100, which were the least preferred formulations. GFB-50 showed intermediate acceptability and did not differ statistically from the most accepted samples.

Preference-based segmentation identified two consumer clusters with distinct liking patterns. Consumers in cluster (1) rated GFB-0, GFB-10, GFB-30, and GFB-C similarly (*p* ≥ 0.05), indicating broad acceptance across low to intermediate PMF levels. In contrast, cluster (2) consumers showed comparable preference (*p* ≥ 0.05) for GFB-50 and GFB-100. Despite these divergent preference profiles, no significant differences were observed between clusters with respect to sociodemographic characteristics (all *p* ≥ 0.05, Table S2).

When acceptance was analyzed according to prior consumption habits, only minor differences emerged between consumers who reported consuming GFB (Yes) and those who did not (No). In both groups, GFB-C and GFB-30 received the highest acceptance scores. However, Yes-consumers assigned significantly lower scores to GFB-100 than No-consumers, who exhibited greater acceptance of this formulation.

ANOVA results for overall acceptability indicated a significant effect of the BT factor (*p* < 0.001), confirming that PMF incorporation markedly influenced consumer preference among the GFBs. In addition, the cluster factor was also significant (*p* < 0.001), demonstrating that acceptability varied according to distinct consumer preference profiles. In contrast, the prior consumption habits factor did not significantly affect overall acceptability (*p* ≥ 0.05), suggesting that liking was largely independent of whether participants routinely consumed gluten-free bread. Notably, significant interaction effects were observed between BT and CL (*p* < 0.001) and between BT and CH (*p* = 0.0038).

**Figure 6:**
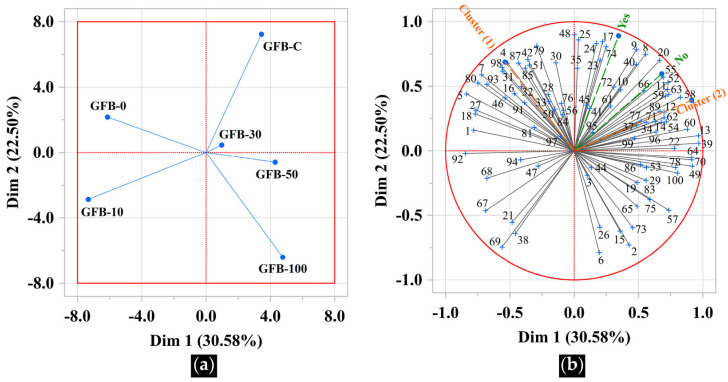
Internal preference map. (**a**) Gluten-free breads (GFBs) as affected by whole-grain pearl millet flour incorporation (PMF, %). C: commercial; (**b**) consumers segmented by preference profile (cluster (1); cluster (2)) and by prior consumption (Yes; No).

Internal preference mapping revealed clear differentiation among bread formulations based on consumer acceptance (Figure 6a). The first two dimensions explained 53.08% of the total variability (Dim1 = 30.58%; Dim2 = 22.50%). GFB-C, positioned in the upper right quadrant, was clearly separated from the experimental breads, particularly along Dim1, indicating a distinct acceptance pattern. This separation likely reflects differences in ingredient composition and/or industrial processing conditions.

Among experimental breads, GFB-0 and GFB-10 clustered closely, suggesting that low PMF incorporation preserves sensory characteristics comparable to the base formulation. In contrast, GFB-30 and GFB-50 were located near the center of the map, reflecting an intermediate acceptance profile between low and high PMF levels. GFB-100, positioned farther away in the lower right quadrant, exhibited the most distinct profile among the experimental breads, indicating that complete replacement of the BF substantially altered consumer acceptance.

Figure 6b further illustrates the segmentation of consumers into two clearly differentiated preference clusters. Cluster (1) was distributed on the left side of the map, indicating a preference for formulations with low PMF incorporation, particularly GFB-0 and, to a lesser extent, GFB-10. The concentration of cluster (1) in the upper left quadrant reflects its close association with the base formulation, suggesting that minimal PMF addition preserves sensory attributes favored by this group. In contrast, cluster (2) was predominantly located on the right side of the map and was associated with formulations containing higher PMF levels, notably GFB-50 and GFB-100, as well as with GFB-C. Most consumers in this cluster were positioned in the upper right quadrant, where GFB-C and, to a lesser extent, GFB-30 were located, indicating broader acceptance of formulations with more pronounced compositional changes. Notably, this quadrant included consumers from both prior gluten-free bread consumption segments (Yes and No), suggesting that preference for these samples was not strongly conditioned by consumption habits. Only a small number of consumers were oriented toward the lower right quadrant, associated with GFB-100, indicating that complete substitution with PMF appealed to a limited subset of participants.

## 4. Conclusions

Starch-based breads, a major category of commercial gluten-free products, were improved by incorporating whole-grain pearl millet flour (PMF). The sequential milling and sieving strategy reduced insoluble fiber size while preserving starch granule integrity, limiting incompatibilities with starch-protein polymers during processing and enabling nutritional enrichment without loss of bread-like functionality. PMF incorporation systematically modified pasting behavior and dough viscoelasticity (G′, G″), which translated into changes in loaf volume, texture, color, and consumer perception. Microstructurally, increasing PMF shifted the system from starch continuity toward fiber–protein-dominated matrices, producing smoother cell walls, darker crumbs, and a transition from larger gas cells at low to intermediate levels to compact structures at high substitutions. Unexpectedly, moderate incorporation enhanced dough expansion and gas retention, whereas excessive PMF promoted premature rigidity, higher firmness, and lower liking, while minimal substitution yielded modest nutritional improvement. Thus, starch acted as the primary scaffold, and fibers and proteins behaved as concentration-dependent interfacial regulators of bubble stability. Among formulations, 30% PMF achieved the best balance, improving protein and dietary fiber contents while maintaining acceptable specific volume, crumb texture, and overall acceptability comparable to commercial gluten-free bread. These results identify 30% PMF as a technologically viable route to nutritionally superior gluten-free bakery products.

## Figures and Tables

**Table 1:** Overall acceptance ^§^ of gluten-free breads and according to participants’ segmentation by profile and consumption habits.

Bread Type (BT)	Participants																				
	Overall				By Preference Segment									By Prior Consumption Habit							
	*n* = 100				Cluster (1) *n* = 53				Cluster (2) *n* = 47					Yes *n* = 42				No *n* = 58			
GFB-0	5.8	±	2.10	*bc*	6.6	±	1.70	*abA*	4.8	±	2.10	*cdB*		5.5	±	1.80	*bcA*	5.9	±	2.30	*bA*
GFB-10	5.7	±	2.60	*c*	7.3	±	1.50	*aA*	3.9	±	2.30	*dB*		5.7	±	2.70	*bcA*	5.6	±	2.50	*bA*
GFB-30	6.8	±	2.00	*a*	7.3	±	1.60	*aA*	6.3	±	2.30	*abB*		6.9	±	2.00	*aA*	6.8	±	1.10	*aA*
GFB-50	6.3	±	2.30	*ab*	6.2	±	2.20	*bA*	6.5	±	2.40	*abA*		6.5	±	2.40	*abA*	6.2	±	2.20	*abA*
GFB-100	5.6	±	2.50	*c*	5.3	±	2.50	*cA*	5.8	±	2.50	*bcA*		4.8	±	2.60	*cB*	6.1	±	2.20	*abA*
GFB-C	6.9	±	2.00	*a*	6.6	±	2.10	*abA*	7.2	±	1.80	*aA*		7.1	±	1.78	*aA*	6.8	±	2.20	*aA*

^§^ Evaluated on a structured 9-point hedonic scale varying from (1) dislike extremely to (9) like extremely. GFB: gluten-free bread. C: commercial. Mean values ± standard deviations with the same lowercase [or uppercase] letters within the same column [or row in a preference segment or consumption habit] do not differ significantly (*p* ≥ 0.05) according to Tukey’s test [or Student’s *t*-test].

## Data Availability

The original contributions presented in this study are included in the article/supplementary material. For more information, please contact the corresponding authors.

## Supplementary Materials

The following supporting information can be downloaded at: https://www.mdpi.com/article/10.3390/foods15050926/s1, Table S1: Proximate composition and instrumental color of experimental breads and wheat bread; Table S2: Sociodemographic characteristics of the study participants, according to consumer preference clusters and prior gluten-free bread (GFB) consumption. Table S3. Pasting and hydration responses of a starch-based flour blend as affected by whole-grain pearl millet flour (PMF) incorporation, used in Figure 2. Table S4. Rheological properties, dough and bread specific volume, and moisture distribution responses of a starch-based dough as affected by whole-grain pearl millet flour (PMF) incorporation, used in Figure 2.

## Abbreviations

The following abbreviations are used in this manuscript:

PMF	Whole-grain pearl millet flour
BF	Base flour
BRM	Breaking roller mill
RRM	Reduction roller mill
HM	Hammer mill
D	Discarded fraction
F	Fibrous fraction
P	Milled product
PT	Pasting temperature
PV	Peak viscosity
TV	Trough viscosity
GV	Gelation viscosity
BV	Breakdown viscosity
SV	Setback viscosity
WSI	Water solubility index
WAI	Water absorption index
MD	Starch granule mean diameter
G′	Elastic modulus
G″	Viscous modulus
DSV	Dough specific volume
BSV	Bread specific volume
HA	Crumb hardness
CO	Crumb cohesiveness
SP	Crumb springiness
CH	Crumb chewiness
BT	Bread type
GFB	Gluten-free bread
GFB-C	Commercial gluten-free bread
WB	Wheat bread
ANOVA	Analyses of variance
PCA	Principal component analysis
