# Supplementary material for: Enhancing Gluten-Free Bread Quality with Whole-Grain Pearl Millet Flour: A Physicochemical and Sensory Approach
Source: Foods. 2026 Mar 6;15(5):926. doi: 10.3390/foods15050926 (PMC12984801; doi:10.3390/foods15050926)
Supplement: Supplementary file 1 [file foods-15-00926-s001.zip › foods-4138247-supplementary.pdf]

foods

MDPI

Table S1. Proximate composition and instrumental color of experimental breads and wheat bread.

Component [1]	Experimental gluten-free breads (GFBs) with varying PMF [2] substitution levels (%)					Wheat bread
	GFB-0	GFB-10	GFB-30	GFB-50	GFB-100	WB
	Proximate composition (%, dry basis) [3]					
ASH	1.76 ± 0.01 *e*	1.86 ± 0.02 *d*	2.06 ± 0.03 *c*	2.29 ± 0.03 *b*	2.81 ± 0.00 *a*	1.75 ± 0.09
PRO	3.47 ± 0.00 *e*	4.14 ± 0.08 *d*	5.65 ± 0.16 *c*	7.25 ± 0.20 *b*	11.02 ± 0.00 *a*	12.18 ± 0.08
LIP	1.86 ± 0.14 *d*	2.78 ± 0.28 *cd*	3.96 ± 0.55 *bc*	4.73 ± 0.26 *b*	6.29 ± 0.37 *a*	2.49 ± 0.06
DF	7.65 ± 0.66 *e*	9.83 ± 0.33 *d*	12.54 ± 0.16 *c*	14.36 ± 0.01 *b*	18.24 ± 0.11 *a*	5.62 ± 0.42
SDF	1.61 ± 0.32 *b*	1.80 ± 0.02 *ab*	2.05 ± 0.17 *ab*	2.17 ± 0.26 *ab*	2.53 ± 0.12 *a*	1.70 ± 0.08
IDF	6.04 ± 0.34 *e*	8.03 ± 0.35 *d*	10.50 ± 0.00 *c*	12.19 ± 0.27 *b*	15.71 ± 0.01 *a*	3.92 ± 0.33
CHOa	85.26 ± 0.79 *a*	81.39 ± 0.01 *b*	75.78 ± 0.52 *c*	71.37 ± 0.50 *d*	61.64 ± 0.27 *e*	77.96 ± 0.36
	Instrumental color [3]					
L*	68.90 ± 0.12 *a*	64.80 ± 0.04 *b*	64.53 ± 0.01 *c*	62.74 ± 0.01 *d*	56.96 ± 0.05 *e*	75.70 ± 0.03
a*	-0.58 ± 0.04 *e*	-0.44 ± 0.01 *d*	0.02 ± 0.01 *c*	0.46 ± 0.03 *b*	1.21 ± 0.04 *a*	0.50 ± 0.03
b*	4.93 ± 0.31 *d*	7.35 ± 0.02 *c*	10.47 ± 0.01 *b*	11.95 ± 0.05 *a*	11.44 ± 0.10 *a*	17.35 ± 0.03

[1] ASH: ashes, PRO: protein, LIP: lipids, DF: dietary fiber, SDF: soluble dietary fiber, IDF: insoluble dietary fiber, CHOa: available carbohydrates, L*: lightness, a*: red-green coordinate, b*: yellow-blue coordinate. [2] PMF: whole-grain pearl millet flour. [3] Means (n = 4 for proximate composition; n = 8 for instrumental color) ± standard deviations within a row followed by the same lowercase letter are not significantly different ($p \geq 0.05$) according to Tukey's test.

Table S2. Sociodemographic characteristics of the study participants, according to consumer preference clusters and prior gluten-free bread (GFB) consumption.

Characteristics	Participants						
	Overall	By preference profile			By prior consumption habit		
	n = 100	Cluster (1) n = 53	Cluster (2) n = 47	p-value	Yes n = 42	No n = 58	p-value
Genre							
Female	57	28	29	0.37 ns	33	24	0.98 ns
Male	43	25	18		25	18	
Age							
18-25	28	16	12	0.53 ns	13	15	0.01 **
26-35	34	15	19		15(-)*	19(+)*	
36-45	21	11	10		17(+)*	4(-)*	
> 46	17	11	6		13	4	
Scholarly							
Elementary School	2	1	1		1	1	0.14 ns
High School	20	12	8		15	5	
Higher Education	49	24	25		23	26	
Postgraduate Studies	29	16	13		19	10	
Income (R$)							
< 1.518,00	22	11	11	0.40 ns	11	11	0.02 *
> 1.518,00 to 3.036,00	26	16	10		12	14	
> 3.036,00 to 7.590,00	17	10	7		10	7	
> 7.590,00 to 15.180,00	10	7	3		8	2	
> 15.180,00	17	6	11		15(+)**	2(-)**	
I prefer not to answer	8	3	5		2(-)*	6(+)*	

(+) or (-) indicates an observed value that is greater or less than the expected theoretical value. *** p < 0.001, ** p < 0.01, and * p < 0.05; chi-square effect per cell.

https://doi.org/10.3390/xxxxx

Table S3. Pasting and hydration responses of a starch-based flour blend as affected by whole-grain pearl millet flour (PMF) incorporation, used in Figure 2.

Property [1]	Gluten-free flours with varying PMF [2] substitution levels (%)					Mean value
	0	10	30	50	100	
			Pasting properties [3]			
IV	43.5 ± 4.9 *a*	58.0 ± 8.49 *a*	42.5 ± 12.0 *a*	34.5 ± 7.8 *a*	-2.0 ± 0.0 *b*	35 ± 22.5
t-PT	5.40 ± 0.00 *ab*	5.43 ± 0.05 *a*	5.50 ± 0.05 *a*	5.27 ± 0.00 *b*	5.43 ± 0.05 *a*	5.4 ± 0.09
PT	70.38 ± 0.32 *ab*	71.35 ± 0.42 *ab*	71.83 ± 0.53 *a*	68.80 ± 0.21 *c*	70.18 ± 0.25 *bc*	71 ± 1.17
PV	2565 ± 29.7 *a*	884 ± 30.4 *b*	658 ± 5.7 *c*	690 ± 29.7 *c*	741 ± 14.1 *c*	-
TV	2421 14.1 *a*	690 ± 27.6 *b*	348 ± 2.8 *c*	295 ± 14.8 *cd*	252 ± 4.2 *d*	-
GV	6379 ± 110 *a*	2308 ± 59.4 *b*	1496 ± 5.7 *c*	1399 ± 56.6 *c*	1354 ± 36.1 *c*	-
BV	144 ± 15.6 *e*	194 ± 2.8 *d*	310 ± 8.5 *c*	396 ± 14.8 *b*	489 ± 9.9 *a*	-
SV	3958 ± 95.5 *a*	1619 ± 31.8 *b*	1148 ± 8.5 *c*	1105 ± 41.7 *c*	1102 ± 31.8 *c*	-
			Hydration properties [3]			
WSI	0.90 ± 0.09 *e*	1.52 ± 0.10 *d*	2.60 ± 0.04 *c*	4.45 ± 0.10 *b*	11.15 ± 0.11 *a*	-
WAI	1.75 ± 0.05 *a*	1.62 ± 0.05 *a*	1.60 ± 0.06 *a*	1.52 ± 0.04 *a*	1.23 ± 0.08 *b*	-

[1] IV: initial viscosity (mPa·s), *t*-PT: pasting onset (min), PT: pasting temperature (°C), PV: peak viscosity (mPa·s), TV: trough viscosity (mPa·s), GV: gelation viscosity (mPa·s), BV: breakdown viscosity (mPa·s), SV: setback viscosity (mPa·s), WSI: water solubility index (g soluble solids per 100 g of flour on a dry basis), WAI: water absorption index (g absorbed water per g of insoluble solids in the flour). [2] PMF: whole-grain pearl millet flour. [3] Means (n = 4 for pasting properties; n = 8 for hydration properties) ± standard deviations within a row followed by the same lowercase letter are not significantly different ($p \geq 0.05$) according to Tukey's test.

https://doi.org/10.3390/xxxxx

Table S4. Rheological properties, dough and bread specific volume, and moisture distribution responses of a starch-based dough as affected by whole-grain pearl millet flour (PMF) incorporation, used in Figure 2.

Property [1]	Experimental gluten-free products with varying PMF [2] substitution levels (%)					Wheat bread
	0	10	30	50	100	WB
	Viscoelastic properties of doughs before fermentation (kPa) [3]					
G′ at 0.1 Hz	92.0 ± 3.7 *aA*	12.8 ± 0.71 *cA*	9.6 ± 0.7 *cA*	8.6 ± 0.3 *cA*	11.8 ± 1.5 *bA*	-
G″ at 0.1 Hz	21.51 ± 0.47 *aB*	3.35 ± 0.25 *cB*	2.64 ± 0.04 *cB*	2.45 ± 0.10 *cB*	3.51 ± 0.46 *bB*	-
G′ at 10 Hz	168.3 ± 3.3 *aA*	20.0 ± 1.44 *cA*	17.8 ± 0.8 *cA*	23.6 ± 3.0 *cA*	42.5 ± 2.2 *bA*	-
G″ at 10 Hz	36.32 ± 0.69 *aB*	4.80 ± 0.18 *cB*	4.35 ± 0.15 *cB*	5.53 ± 0.57 *cB*	10.00 ± 0.59 *bB*	-
	Specific volume of doughs and breads (cm^3/g) [3]					
DSV_{onset}	0.76 ± 0.03 *cγ*	0.78 ± 0.01 *bcδ*	0.81 ± 0.01 *bcδ*	0.84 ± 0.00 *bδ*	0.94 ± 0.02 *aδ*	1.03 ± 0.03
$DSV_{20\ min}$	0.89 ± 0.05 *dγ*	0.94 ± 0.01 *cdγ*	1.01 ± 0.00 *bcγ*	1.04 ± 0.00 *bγ*	1.31 ± 0.01 *aγ*	1.41 ± 0.01
$DSV_{40\ min}$	1.08 ± 0.05 *dβ*	1.16 ± 0.02 *cdβ*	1.26 ± 0.01 *bcβ*	1.30 ± 0.01 *bβ*	1.58 ± 0.04 *aβ*	1.93 ± 0.03
DSV_{end}	1.40 ± 0.04 *cα*	1.58 ± 0.02 *bα*	1.64 ± 0.02 *abα*	1.58 ± 0.03 *bα*	1.73 ± 0.04 *aα*	2.25 ± 0.01
FT (min)	65	65	60	60	55	60
BSV	2.40 ± 0.06 *b*	2.68 ± 0.05 *a*	2.56 ± 0.07 *ab*	2.41 ± 0.03 *b*	2.13 ± 0.04 *c*	3.02 ± 0.03
OS (%)	71.4	69.1	56.6	52.4	23.1	33.8
	Moisture distribution of breads (%) [3]					
Whole bread	47.89 ± 0.38 *a*	47.31 ± 0.54 *ab*	45.37 ± 0.83 *bc*	43.35 ± 0.16 *cd*	42.24 ± 0.71 *d*	-
Crumb	52.43 ± 0.04 *a*	51.80 ± 0.19 *a*	51.33 ± 0.80 *a*	50.75 ± 0.70 *a*	48.25 ± 0.75 *b*	-
Crust	35.88 ± 0.01 *a*	29.88 ± 0.76 *b*	25.92 ± 0.04 *c*	24.34 ± 0.41 *c*	23.48 ± 1.37 *c*	-

[1] G′: elastic modulus, G″: viscous modulus, DSV: dough specific volume. Subscripts: onset (before starting fermentation), 20 (after 20 min), 40 (after 40 min), end (after fermentation is complete), FT: fermentation time at the end, BSV: bread specific volume. OS: oven spring (OS = $100\% \times (BSV - DSV_{end})/DSV_{end}$. [2] PMF: whole-grain pearl millet flour. [3] Means (n = 4 for viscoelastic properties; n = 6 for specific volumes and moisture contents) ± standard deviations within a row followed by the same lowercase letter, or within a column followed by the same uppercase letter for viscoelastic properties or by the same Greek letter for DSV, are not significantly different (p ≥ 0.05) according to Tukey's test.
